# Characterization of the *XTH* Gene Family: New Insight to the Roles in Soybean Flooding Tolerance

**DOI:** 10.3390/ijms19092705

**Published:** 2018-09-11

**Authors:** Li Song, Babu Valliyodan, Silvas Prince, Jinrong Wan, Henry T. Nguyen

**Affiliations:** 1 Institutes of Agricultural Science and Technology Development, Joint International Research Laboratory of Agriculture and Agri-Product Safety, Co-Innovation Center for Modern Production Technology of Grain Crops, Yangzhou University, Yangzhou 225009, China; 2 National Center for Soybean Biotechnology and Division of Plant Sciences, University of Missouri, Columbia, MO 65211, USA; valliyodanb@missouri.edu (B.V.); sjprince@noble.org (S.P.); wanj@missouri.edu (J.W.); 3 Noble Research Institute, 2510 Sam noble Pkwy, Ardmore, OK 73401, USA

**Keywords:** *Glycine max*, *XTH* gene family, transgenic soybean, plant genome, plant hormone, flooding, root plasticity

## Abstract

Xyloglucan endotransglycosylases/hydrolases (XTHs) are a class of enzymes involved in the construction and remodeling of cellulose/xyloglucan crosslinks and play an important role in regulating cell wall extensibility. However, little is known about this class of enzymes in soybean. Here, 61 soybean *XTH* genes (*GmXTH*s) were identified and classified into three subgroups through comparative phylogenetic analysis. Genome duplication greatly contributed to the expansion of *GmXTH* genes in soybean. A conserved amino acid motif responsible for the catalytic activity was identified in all GmXTHs. Further expression analysis revealed that most *GmXTHs* exhibited a distinct organ-specific expression pattern, and the expression level of many *GmXTH* genes was significantly associated with ethylene and flooding stress. To illustrate a possible role of *XTH* genes in regulating stress responses, the *Arabidopsis* *AtXTH31* gene was overexpressed in soybean. The generated transgenic plants exhibited improved tolerance to flooding stress, with a higher germination rate and longer roots/hypocotyls during the seedling stage and vegetative growth stages. In summary, our combined bioinformatics and gene expression pattern analyses suggest that *GmXTH* genes play a role in regulating soybean stress responses. The enhanced soybean flooding tolerance resulting from the expression of an *Arabidopsis* XTH also supports the role of *XTH* genes in regulating plant flooding stress responses.

## 1. Introduction

Xyloglucan endotransglycosylases/hydrolases (XET/XTHs also named XTHs) are classified as glycoside hydrolase family 16 (available online: http://www.cazy.org/fam/GH16.html) and play an important role in organ elongation by modifying xyloglucan chains or catalyzing the hydrolysis of xyloglucan [1,2,3,4]. Several studies have emphasized the significant role of this gene family in the regulation of cell wall extensibility. Overexpression of the *BcXTH1* gene from *Brassica campestris* enhanced stem elongation in *Arabidopsis* by promoting cell expansion and elongation [5]. Increased *SlXET1* activity affects the xyloglucan structure during the fruit ripening and softening process in transgenic tomato fruit [6]. Overexpression of *GhXTH1* in cotton loosens and elongates cell wall fibers due to cleavage down the xyloglucan-cellulose chains [7,8]. *PtxtXET16-34* is strongly expressed in primary-walled xylem. Transgenic hybrid Aspen analysis indicated that wood cell expansion and xyloglucan content were affected when the *PtxtXET16-34* gene was overexpressed [9]. *AtXTH31* regulates cell wall xyloglucan content, and *AtXTH21* influences the development of primary roots by regulating the deposition of cellulose and the thickness of the cell wall in *Arabidopsis* [10,11].

It was reported that the XTH enzyme activity may vary with changes in environmental conditions (i.e., abiotic stresses), and that hormones play important roles in tuning XTH activity during plant development. XET activity in the maize primary root elongation zone contributes to cell wall loosening at low water potential, which is partly regulated by abscisic acid (ABA) [12,13]. Low water potential decreased the XET activity in the hypocotyl elongation zone of dark-grown soybean [14]. A decrease in XET activity was also reported in the basal 5–10 mm of maize primary roots treated with polyethylene glycol solution, which reduced the cell wall extensibility and cell elongation in that region [15]. *CaXTH3* overexpressing transgenic *Arabidopsis* showed improved tolerance to water deficit and less tolerance to high salinity compared to wild type [16]. *OsXTH8*, which is highly expressed in the vascular bundles of leaf sheath and young nodal roots in rice, was upregulated by gibberellic acid. Repressed growth in transgenic rice was associated with knocking down the expression of *OsXTH8* [17]. Loss of function of *AtXTH31* reduces sensitivity to ABA and faster germination in *Arabidopsis* [18]. Downregulation of *XTH8* and *XTH31* is also responsible for the reduced leaf cell expansion of the *Arabidopsis siz1* mutant in an SA-dependent manner [19]. The homologue of *GmXTH16* in maize is induced by flooding and ethylene and is associated with aerenchyma development [20].

As the third most cultivated crop worldwide, soybean provides protein, oil, and plant natural products for human and animal consumption, but its production is limited by environmental constraints [21,22,23]. Among the major abiotic stresses, soybean is particularly sensitive to flooding stress, as plant growth and grain yields are markedly reduced in flooded soil [24]. The downregulation of gene expression related to cell wall metabolism, cellulose synthesis, and cell wall degradation caused by flooding indicates that cell wall biosynthesis is inhibited by flooding [25]. Therefore, functional characterisation of the *XTH* gene family in soybean will be very useful for revealing the mechanism of soybean flooding resistance. However, little is known about soybean *XTH*s, except for the report showing that *pBRU1* (*GmXTH16*) is involved in brassinosteroid-regulated soybean epicotyls elongation [26]. The availability of soybean genome sequences and comparative analysis of the XTH gene family across plant species provide an excellent opportunity to explore the XTH diversity in soybean. In this study, genome-wide analyses of soybean *XTH*s were performed, including phylogenetic analysis, chromosomal distribution, structural, and conserved motif (DExDxEFLG) analysis. Then, a comparative analysis of the XTH gene family transcriptome in soybean tissues was performed. Further functional validation of the roles of *AtXTH31* was executed in transgenic soybean during the early seedling stage under flooding stress.

## 2. Results

### 2.1. Genome-Wide Identification of Soybean XTH Family and Phylogenetic Relationship

A total of 61 *GmXTHs* were identified with a blasting core value over 100 by using the AtXTH31 amino acid sequence as query. They were designated *GmXTH1* through *GmXTH61* according to their positions on chromosomes 1 to 20 (Supplemental Table S1). The putative proteins encoded by these *GmXTHs* document the conserved structural features of the XTHs.

**Figure 1:**
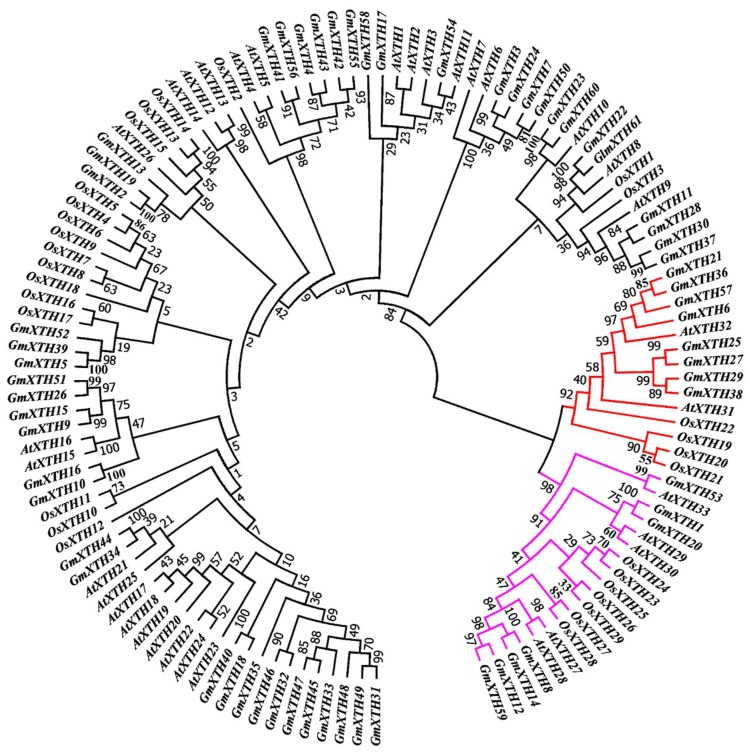
An unrooted phylogenetic tree for *AtXTH*, *OsXTH*, and *GmXTH* genes. A phylogenetic tree was constructed using the neighbor-joining method implemented in MEGA7. The number beside the branches represents bootstrap values based on 1000 replications. The XTH members are classified into three subfamilies. Genes from groups I/II and III are shown in the black and red/pink lines, respectively. Group III was designated group IIIA (red) and group IIIB (pink).

An unrooted phylogenetic analysis was constructed using a total of 123 full-length XTH protein sequences from *Arabidopsis* (AtXTH), rice (OsXTH), and soybean (GmXTH). According to the NJ phylogenetic tree, 46 *GmXTH* genes were clustered in group I/II, and 15 members were classified in group III (Figure 1). Two major groups have been previously classified based on the sequence similarity as group I/II and group III in *Arabidopsis* and rice [27,28]. Phylogenetic analysis revealed that the soybean *GmXTH* gene family was expanded widely in contrast to *Arabidopsis* and rice, which may correspond to the larger genome of soybean. Group III is further divided into two subgroups: group IIIA (red) and group IIIB (pink), which contains eight and seven *GmXTH* genes, respectively.

### 2.2. Chromosomal Location and Duplication Process of GmXTHs

**Figure 2:**
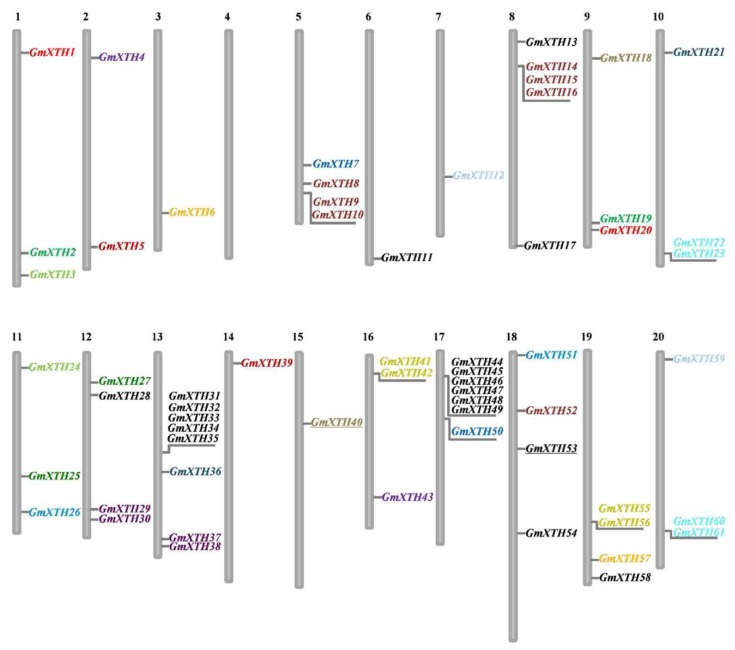
Chromosomal location of 61 *GmXTH* genes along soybean’s 20 chromosomes. Physical map showed the distribution of the *Glycine max XTH* genes along the 20 chromosomes with colors indicating duplicated gene pairs. The chromosome number is indicated at the bottom.

Genome localization analysis revealed that *GmXTHs* were widely dispersed across 19 of the 20 chromosomes (Figure 2). Most *GmXTH* genes were distributed on the chromosome arms, except for two genes (*GmXTH40* and *GmXTH53*) which are located in the heterochromatin regions around the centromeric repeats. Chromosome 13 contained the largest number (eight *XTH* genes), followed by seven on chromosome 17 and five on chromosome 8. No *XTH* genes were found on chromosome 4, and the remaining chromosomes each contained one to four *XTH* genes.

**Figure 3:**
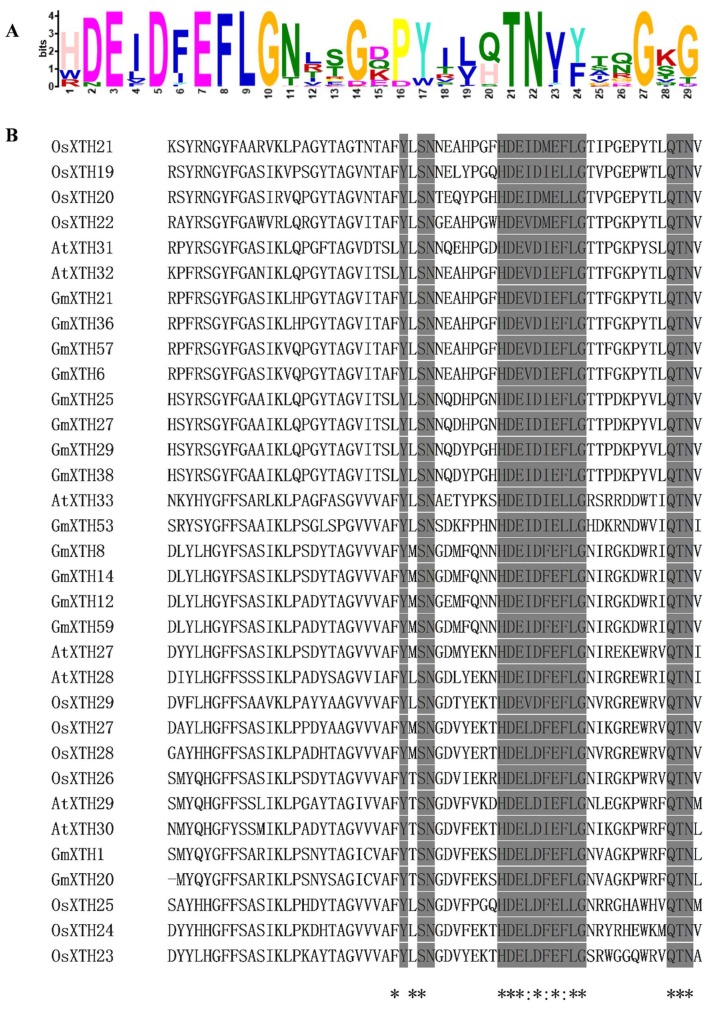
Conserved protein motifs in soybean XTHs. (**A**) Motifs in XTH protein sequences of 61 GmXTH identified with the MEME tool. (**B**) Alignment of the putative-site amino acid residues in group III XTH proteins from *Arabidopsis*, rice, and soybean constructed with the CLUSTALW2 program. Amino acid residues that are identical within this motif are indicated by gray shading. “*” means that the residues or nucleotides in that column are identical in all sequences in the alignment. “:” means that conserved substitutions have been observed.

Here, we found that tandem duplication or segmental duplication was involved in the expansion of the *GmXTH* gene family (Supplementary Table S3). Mapping the *XTH* genes to their chromosome physical positions (Figure 3) revealed that many *XTH* genes were clustered together, suggesting that they might be the result of tandem duplication events. For example, *GmXTH31*, *32*, *33*, *34*, and *35* on chromosome 13 and *GmXTH44*, *45*, *46*, *47*, *48*, and *49* on chromosome 17 come from tandem duplication. *GmXTH41*/*42* pair and *GmXTH55*/*56* pair come from tandem duplication. In addition, most of the segmental duplications of *GmXTH* genes occurred approximately 13 Million years ago when Glycine-specific duplication occurred in the soybean genome (Schmutz et al., 2010, [29] Supplementary Table S3). The Ka/Ks ratio for each segmentally duplicated gene pair varied from 0.06 to 0.46. This analysis suggests that all mutations in paralogous *GmXTH* genes are neutral or disadvantageous, as their Ka/Ks ratios were less than 1. In general, tandem and segmental duplication contributed to *XTH* gene expansion in soybean. Only seven genes (*GmXTH11*, *GmXTH13*, *GmXTH17*, *GmXTH28*, *GmXTH53*, *GmXTH54*, and *GmXTH58*) were not involved in duplication events.

### 2.3. Gene Structure and Conserved Protein Motif Analysis

To gain further insights into the evolutionary relationships among *GmXTH* genes, the exon/intron structures were predicted based on the alignment of coding sequence (CDS) sequences with corresponding genomic DNA sequences. As illustrated in Supplementary Figure S1, all members of the *GmXTH* family contain three or four exons. Several genes showed a different structure; for example, *GmXTH44* has an extremely long 5′ untranslated region (UTR), and *GmXTH39*, *GmXTH5*, and *GmXTH58* have no 5′ and 3′ UTRs.

All GmXTH proteins contained the conserved amino acid motif DExDxEFLG (Figure 3A), which is predicted to be responsible for the catalytic activity [30,31]. Therefore, GmXTHs reported here may cut/rejoin xyloglucan chains or catalyze the hydrolysis of xyloglucan. Compared with the genes of group III, this conserved motif could be extended upstream and downstream with a more conserved motif among *Arabidopsis*, rice, and soybean (Figure 3B).

### 2.4. GmXTHs Show an Organ-Specific Expression Pattern

**Figure 4:**
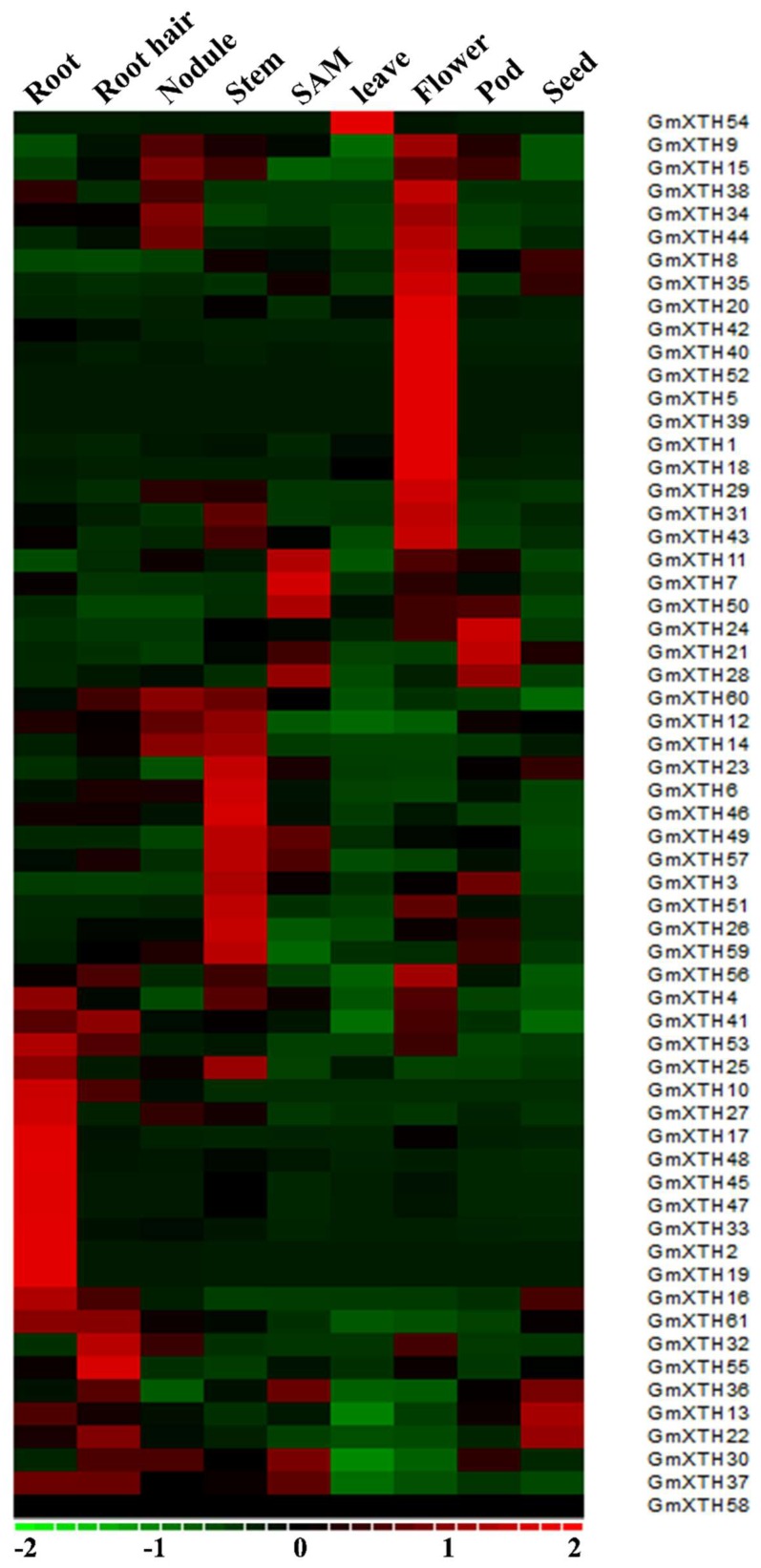
Heatmap of the expression profiles of the *GmXTH* gene family in nine organs. Relative organ expression levels of *GmXTHs* based on RNA-seq data were used to construct the heatmap. The expression values (Reads Per Kilobase Million) were median-cantered and normalized for each gene in different tissues before transforming to color scale. The color bar at the bottom shows the range of expression values from highest expression level (red) to lowest expression level (green), and 0 is the median expression level (Black). SAM (Shoot Apical Meristem).

To determine the expression patterns of *GmXTH* genes, we used publicly available genome-wide transcriptome data of soybean organs as a resource [32]. As shown in Figure 4, *GmXTH* genes are broadly expressed in various soybean organs. However, most of the *GmXTHs* exhibit distinct tissue-specific expression patterns. For example, fourteen *GmXTHs* (*XTH4*, *41*, *53*, *10*, *27*, *17*, *48*, *45*, *47*, *33*, *2*, *19*, *16*, and *61*) were highly expressed in roots, eighteen *GmXTHs* (*XTH9*, *38*, *34*, *44*, *8*, *35*, *20, 42, 40*, *52*, *5*, *39*, *1*, *18*, *29*, *31*, *43*, and *56*) were highly expressed in flowers, thirteen *GmXTHs* (*XTH60*, *12*, *14*, *23*, *6*, *46*, *49*, *57*, *3*, *51*, *26*, *59*, and *25*) were highly expressed in stems, and three *GmXTHs* (*XTH 36*, *13*, and *22*) highly expressed in seeds, whereas expression levels were relatively low in other organs. In contrast, several organs only contained one or a few genes that showed specific expression patterns. For example, *GmXTH54* was the only gene highly expressed in leaves compared with other organs, and *GmXTH55* was the only gene highly expressed in root hair compared with other organs.

No sequence reads were found for *GmXTH58* in any of the soybean organs included in the study, indicating that *GmXTH58* is probably a pseudogene or expressed under special conditions or at specific developmental stages. No *GmXTH* gene showed specifically higher expression level in nodules. Similar expression patterns were found for some phylogenetically paired genes. For example, *GmXTH25* and *GmXTH27* from group IIIA were highly expressed in roots, and *GmXTH29* and *GmXTH38* from group IIIA were highly expressed in flowers. *GmXTH14*, *GmXTH12,* and *GmXTH59* from group IIIB were highly expressed in stems.

### 2.5. Expression Patterns of GmXTHs Correlated with Flooding Stress

Identification of the regulatory elements indicated that one 4-bp oxygen-deficiency response element (S000493 GTAC) was significantly enriched in most *GmXTH* promoter regions, except *GmXTH23* and *GmXTH55* (Supplementary Table S4). Most *GmXTH* genes contain more than 3 response elements. The main effect of flooding is hypoxia, which reduces submerged plant normal growth and nutrient/water uptake [33]. These results suggest that the *GmXTH* gene family may play important roles during soybean sustained flooding stress.

**Figure 5:**
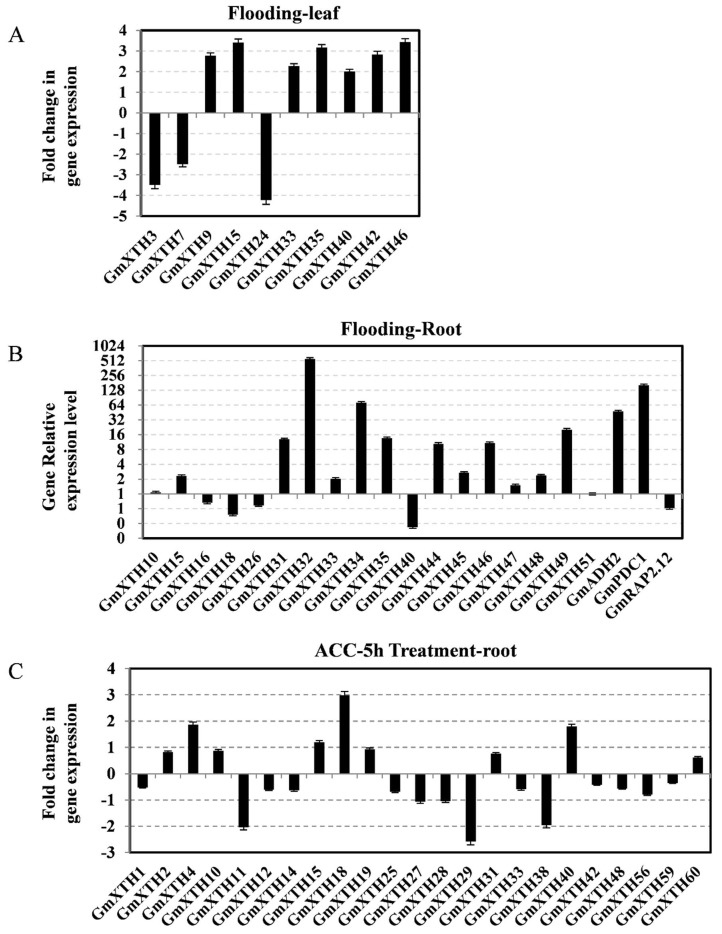
Expression patterns of individual *XTH* genes in response to flooding and ACC treatment studied by qRT-PCR or RNA-seq. (**A**) Expression pattern of individual *XTH* genes in response to 24 h flooding treatment in root and hypocotyl organs of two-day-old soybean seedlings. (**B**,**C**) Organ-specific expression analysis showed that most *XTH* genes were unregulated by the ACC in the root tissue, but there was no significant difference in the aerial parts of the three-week-old soybean. Three flooding-related homologous marker genes in soybean were studied as positive controls. Fold change (Log2) of relative gene expression (Actin (Glyma.18G290800)) of soybean was used as the normalization control.

To further assess whether the expression profiles of *GmXTH* genes are changed under flooding stress, soybean seedlings (Williams 82 genotype) were exposed to flooding conditions for 7 days [34], and their leaves were collected for gene expression analysis by RNA-seq method. Ten out of sixty-one genes were significantly regulated under flooding stress (Figure 5A), and all genes were located in group I/II. Then, the gene expression levels of twenty genes from group I/II were assessed in soybean hypocotyl and root organs under 2 days of flooding stress by quantitative Real-Time PCR (qRT-PCR) method. The results indicated that *GmXTH32* and *GmXTH34*, together with the marker genes GmADH2 and GmPDC1 [35,36], were predominantly upregulated by flooding stress, and all others showed higher expression levels than the untreated control, except four genes that were slightly downregulated (Figure 5B).

Ethylene will quickly accumulate under flooding conditions, and many regulators of submergence respond to ethylene [33,37]. To further investigate the relationship between the expression level of *GmXTH* genes and ethylene, we monitored the abundance of 61 *GmXTH* transcripts in soybean root tissue exposed to 50 μM 1-Aminocyclopropane-1-carboxylic acid (ACC) for 5 h by RNA-seq method (Wang and Nguyen et al., University of Missouri, Columbia, MO. Williams 82, 2015). We found that twenty-three *GmXTH* genes were significantly regulated under ACC treatment (Figure 5). Among them, *GmXTH18* was highly induced, and *GmXTH29* was highly down-regulated. Interestingly, *GmXTH25*, *27*, *28*, and *29* (group IIIA) were downregulated. We also found the *GmXTH40* was oppositely adjusted by flooding in leaf and root tissues. *GmXTH32* and *GmXTH34* were significantly induced by flooding in roots but not in leaves. Interestingly, a similar expression pattern was found for the tandem or segmental duplicated gene pairs located on chr13 and chr17. For example, *GmXTH31* and *GmXTH49* are both upregulated, and *GmXTH18* and *GmXTH40* are both downregulated by flooding stress in the root (Figure 5).

### 2.6. Stable Transgenic Soybean with Overexpression of AtXTH31

**Figure 6:**
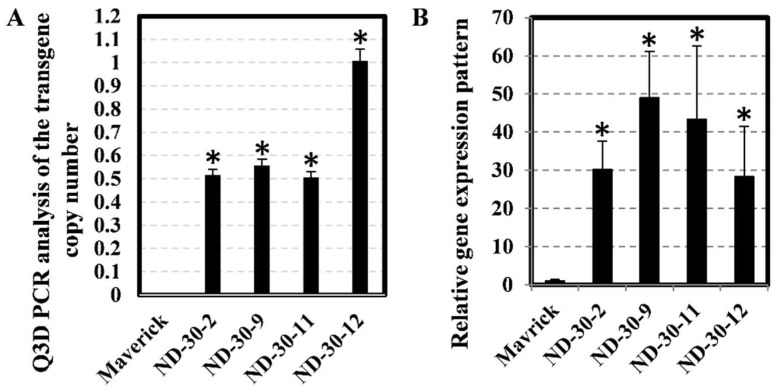
Calculation of transgene AtXTH31 copy numbers and relative expression levels in four transgenic events. (**A**) Ratios of copy number between *AtXTH31* and lectin gene (Glyma.02G009600) were determined by digital PCR in soybean T0 transgenic generation. Soybean transgenic plants contained a single insert copy when the ratio value was equal to 0.5 and two insert copies when the ratio value was equal to 1. (**B**) The relative expression of *AtXTH31* in T3 homozygous transgenic soybean roots determined by qRT-PCR. The relative levels of transcripts were normalized to the soybean actin gene (Glyma.18G290800). Bars represent mean values of three biological replicates (standard error). * indicates significantly different at *p* < 0.05 as tested by Fisher’s least significant difference. Non-transgenic Maverick soybean as a control and MYB2:AtXTH31 transgenic soybean lines ND-30-2, ND-30-9, ND-30-11, and ND-30-12 with overexpression of *AtXTH31* were studied.

To further characterize how *XTH* genes affect root morphology and maintain physiological response under flooding conditions during soybean seedling development, *AtXTH31* (under the control of the AtMyb2 promoter) was transformed into soybean (cultivar Maverick). Over twenty independent transgenic events were generated, and six events were first identified through the general PCR method. Subsequently, the copy number variation of the transgene in those events (T0 generation) was inferred using the Q3D PCR method. Three events (Code: ND-30-2, ND-30-9 and ND-30-11) contained one copy number of the transgene, and one event (Code: ND-30-12) showed two copies of inserts (Figure 6A). However, the other two events showed a very low copy number (less than 0.5), which indicates that those events are chimeras (transgenic plant or plant part that is a mixture of two or more genetically different types of cells). Homozygous T3 transgenic soybean lines from the above events were obtained and confirmed using herbicide-resistance segregation analysis. An ND-30-12 single copy insert line was chosen from segregated T1 generation by the Q3D PCR method.

The transcript abundance of *AtXTH31* in different T3 homozygous transgenic soybean lines was investigated using qRT-PCR. Lines ND-30-2 and ND-30-12 had approximately 28-fold increases compared to the non-transgenic control, whereas lines ND-30-9 and ND-30-11 had moderately high (between 43-fold to 48-fold) increases (Figure 6B).

### 2.7. Transgenic Soybean Exhibits Tolerance to Flooding during the Germination Stage

**Figure 7:**
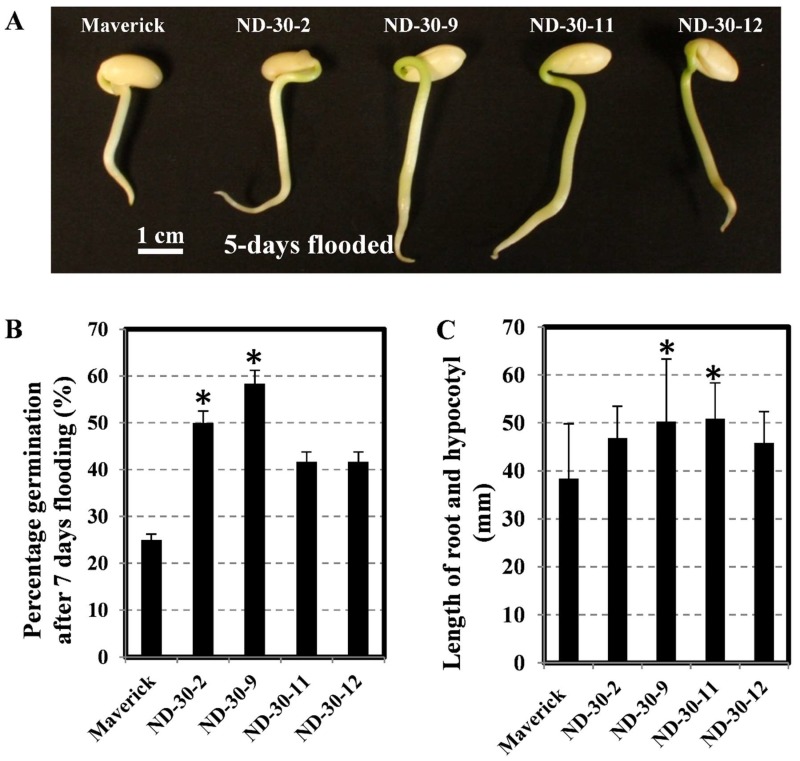
Soybean *AtXTH31* transgenic plants show an enhanced germination ratio and elongated root and hypocotyl under flooding conditions. (**A**) Two-day-old seedlings were flooded with water for 5 days. Bar indicates 1 cm. (**B**) The germination rate of transgenic and wild-type plants under 7 days of flooding. (**C**) Length of roots and hypocotyls of flooded Maverick soybean and transgenic seedlings. (*n* ≥ 30). * indicates differences between the maverick and transgenic soybean (*p* < 0.05).

In a comparison of the flooding tolerance between the control and transgenic lines, the germination rate of each line was counted first. As shown in Figure 7, all transgenic soybean lines had longer roots and hypocotyls (range from 45 mm to 51 mm) than the control (38 mm) after 5 days of flooding (Figure 7A). Four transgenic homozygous T3 transgenic lines with varied transgene expression conferred a range of tolerance with an increase in germination rate after 7 days of flooding, which ranged from 40% to 58% and two lines (ND-30-2 and ND-30-9) showed significant increase (Figure 7B). The length of roots and hypocotyls in the two lines (ND-30-9 and ND-30-11) was significantly greater than that in the control (Figure 7C). These results indicated that overexpressed AtXTH31 in soybean induced higher germination rate, and enhanced root/hypocotyl elongation compared with susceptible parent Maverick.

### 2.8. Transgenic Soybean Exhibits Tolerance to Flooding during the Vegetative Stage

**Figure 8:**
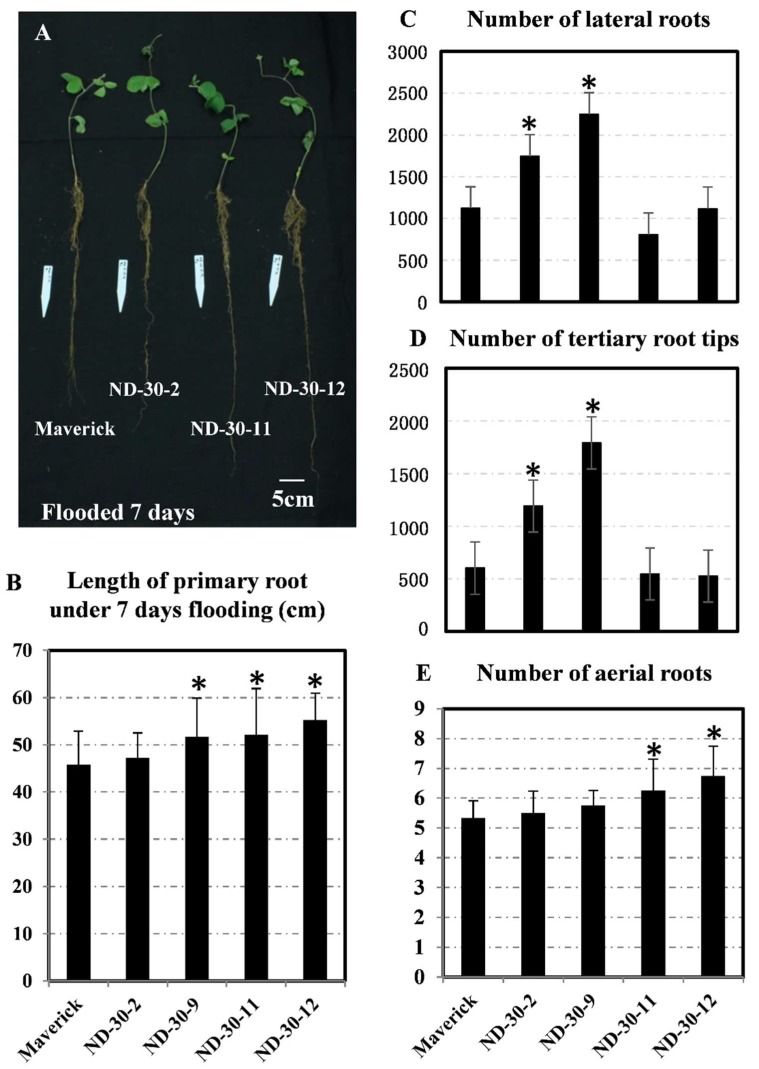
Soybean AtXTH31 transgenic plants show an enhanced flooding tolerance phenotype by promoting adventitious roots, lateral roots, tertiary root tips, and elongated primary roots. (**A**) Flooding effects on soybean seedlings. The V1 stage seedlings were flooded with water for 7 days. Bar indicates 5 cm. (**B**) Length of primary root compared between transgenic and control soybean plants under 7 days of flooding conditions. (**C**–**E**) The effects of flooding on number of lateral roots, tertiary root tips and adventitious roots per transgenic plant. (*n* ≥ 20). * indicates differences between the maverick and transgenic soybean (*p* < 0.05).

Similarly, the ability of transgenic seedlings (V1 stage) to withstand flooding was then investigated. Seedlings were grown up to the V1 stage and flooded for 14 days (Figure 8A). We found that the primary root of transgenic plants was longer than that of non-transgenic controls under flooding conditions. Except for ND-30-2, the roots of all other three transgenic plants showed a significant increase in length (Figure 8B). In addition, two transgenic events (ND-30-2 and ND-30-9) showed great number of lateral roots and tertiary root tips as a response to flooding. Meanwhile, we observed that aerial root formation significantly increased in other two lines than the wild type (Figure 8E). Thus, ectopic expression of AtXTH31 in soybean could promote root development under flooding conditions and provide enhanced tolerance to flooding stress.

## 3. Discussion

The *XTH* gene family has been identified in several plant species, including *Arabidopsis* [27], rice [28], barley [38], poplar [39], tomato [40], and bryophyte [41]. In this study, we report the identification and characterization of the soybean *XTH* gene family and make a comparison to *Arabidopsis* and rice. Expression pattern analysis suggested that *GmXTHs* may play an important role under flooding stress. Transgenic soybean plants overexpressing *AtXTH31* showed an increase in tolerance to flooding, along with the increased aerial root number and elongated primary root length.

### 3.1. Charaterization of GmXTHs Gene Family

Although the role of plant XTHs in regulating cell wall extensibility is well known, there is limited information on the XTH gene family size and the evolutionary relationships between XTH genes in soybean. Previous phylogenetic studies showed that XTHs form three groups in Arabidopsis and rice [27,28]. The number of *GmXTH* genes identified was substantially higher than in *Arabidopsis* and rice, and clustered into three groups. Further, the evolutionary mechanism analysis suggested that GmXTHs family expanded partly due to segmental and tandem duplication events. These duplication events further contributed to the conserved protein motif and gene structure. These *GmXTHs* genes displayed differential expression patterns either between different organs or under flooding stress. For example, *GmXTH22* showed the highest expression level in root hair and seeds, but *GmXTH23* exhibited the highest expression level in stems. *GmXTH29* and *GmXTH30* showed the highest and lower expression levels in flower tissue, respectively. The expression patterns of these paralogous pairs suggest that *GmXTH* gene family might have undergone sub-functionalization or neofunctionalization during the subsequent evolution process. Considering these facts, the characterization of *GmXTH* gene family provides valuable information on the evolution of the *XTH* soybean gene family and underlines basis for future research.

### 3.2. The Expression Patterns of GmXTHs Were Regulated by Flooding and Ethylene

Flooding causes severe production losses in soybean [42,43] through inhibition of stem and root growth, decreased photosynthesis, and induced leaf abscission and premature fruit drop [20,44,45]. Therefore, investigating gene expression patterns of the *GmXTH* gene family can help us advance the fundamental understanding of how soybean adjusts to flooding stress during growth and development.

In this study, we found that the expression pattern of *GmXTH*s may confer precise regulation with temporal, spatial, and environmental conditions. One of the main effects of flooding is the deprivation of oxygen from plant roots, and low oxygen will increase the synthesis of ethylene [46]. Ethylene production was higher in soybean waterlogging-tolerant lines than in susceptible lines [47]. It has been reported that the expression of *XTH* genes is associated with shoot elongation, which is promoted by ethylene in arrowhead tubers [48]. Several *AtXTH* genes were differentially regulated during ACC-induced inhibition of *Arabidopsis* root cell elongation [49]. Ethylene increases *XTH* and *EXPANSIN7* (*EXP7*) expression in *Arabidopsis* roots [50]. Accordingly, investigation of *GmXTH* expression levels under ACC treatment will provide more hints to further gene functional analysis. In this study, we found that 23 *GmXTH* genes were significantly regulated by ethylene in soybean roots, indicating that the hormone ethylene plays important roles in GmXTH-mediated cell wall remodeling during flooding stress. However, further analysis is needed to reveal the relationship between hormone ACC and cell wall remodeling by regulating *XTH* transcription level in soybean.

### 3.3. The Biological Function of AtXTH31 in Soybean Root Development Under Flooding Stress

It was reported that the elongation of soybean roots was suppressed in the first 24 h and then significantly retarded after 48 h under flooding stress, which indicated that the flooding responses in the initial stages are critical for soybean growth and survival [51]. The XTH activity in the hypocotyl elongation zone of dark-grown soybean decreases when the root is exposed to low water potential [14], which directly indicates that XTH may be involved in the abiotic stress response. In this paper, the phenotypes of transgenic soybean plants carrying *AtXTH31* gene on seedling growth under flooding conditions were studied. *AtXTH31* belongs to subgroup IIIA, which was predicted to exhibit hydrolase activity with higher activity in vitro than XET activity [10,52]. *AtXTH31* exhibits a root-specific expression pattern and is involved in *Arabidopsis* cell wall modification and cell elongation [27]. The *xth31* mutant shows slower root elongation [10]. Therefore, the *AtXTH31* gene was selected for heterologous overexpression in soybean. Here we found that transgenic soybean’s ability to produce more adventitious roots and longer primary roots corresponded to an increase in tolerance under flooding stress during early seedling development. It was reported that soybean root length was positively correlated with waterlogging tolerance in soybean germplasm lines [53,54]. Waterlogging-tolerant soybean lines normally developed more adventitious roots than waterlogging-susceptible lines [47,55]. Clearly, our results indicate that XTH-mediated cell wall adjustment may play a critical role in the adaptation of plants to flooding stress, and *AtXTH31* could be a useful candidate gene to develop flooding tolerance lines through molecular transgenic breeding methods. However, the corresponding tolerance mechanisms demand further investigation.

### 3.4. Digital PCR Provides a Simple and Accurate Method for Soybean Transgene Copy Number Analysis

Detection and quantification of transgene copy numbers are very important in characterizing transgenic plants. Recently, the application of digital PCR for the precise analysis of transgene copy numbers in crops has been reported in an array of crops [56,57,58]. This technology accelerates molecular breeding workflow in transgenic plants, enhances data quality in characterizing transgenes, and finally benefits the environment. However, no reports have been available on soybean for detecting copy number variations by the application of digital PCR until now.

In our study, digital PCR technology was applied to validate the transgenic *AtXTH31* copy numbers using T0 plants. Here, the digital PCR method provided more accurate results than those provided by Southern blotting and classical PCR. For example, chimeric plants (a plant or plant part that is a mixture of two or more genetically different types of cells) can be easily identified through the ratio of copy numbers. Furthermore, the dPCR method was continuously used to identify homozygous plants in the T1 generation, which saved the experiment time and no need to evaluate the homozygous lines through calculating the segregation rate of T2. In particular, the dPCR method was successfully applied to choose single-copy insert transgenic lines through analysis of copy number variation in the T1 generation. In summary, the dPCR method provides a very useful technical support for the transgenic soybean research community.

## 4. Materials & Methods

### 4.1. Identification, Chromosomal Location, and Structural Organization of GmXTH Family Members in Glycine Max

All sequence information for genes and proteins was retrieved by searching Phytozome v10.3 database (available online: http://www.phytozome.net) with a BLASTP algorithm using the *AtXTH31* amino acid sequence. The chromosome location of each *GmXTH* was obtained from Phytozome. The exon/intron organizations of *GmXTH*s were visualized with the Gene Structure Display Server program ([59] GSDS: available online: http://gsds.cbi.pku.edu.cn/).

### 4.2. Protein Sequence Alignment, Phylogenetic Analysis, and Gene Duplications of *GmXTH* Genes

Multiple sequence alignments were constructed using ClustalW2 (available online: http://www.ebi.ac.uk/Tools/clustalw2/index.html). Subsequently, a phylogenetic tree was constructed using the neighbor-joining method and implemented using the MEGA7 software tool [60]. The reliability of an inferred tree was confirmed with bootstrap analysis performed with 1000 replications. The evolutionary distances were computed using the Poisson correction method [61] and are in the units of the number of amino acid substitutions per site. A total of 123 coding sequences from *Arabidopsis*, rice, and soybean were collected for phylogenetic analysis. All positions containing gaps and missing data were eliminated. The Ks (synonymous substitutions per synonymous site) and Ka (non-synonymous substitutions per non-synonymous site) values were extracted from the Plant Genome Duplication Database (PGDD: available online: http://chibba.agtec.uga.edu/duplication/), and these were used for calculating the approximate dates of duplication events. The date of duplication events was subsequently estimated according to the equation T = Ks/2λ, in which the mean synonymous substitution rate (λ) for soybean is 6.1 × 10^−9^ [62].

### 4.3. Plant Growth, Hormonal/Flooding Treatments, and Tissue Collection

Soybean cultivar Williams 82 was used for gene expression pattern analysis. For ethylene precursor 1-aminocyclopropane-1-carboxylic acid (ACC) treatment, plants were grown in 4-gallon pots (Greenhouse megastore, USA) containing a 3:1 mixture of turface and sand in a growth chamber under the conditions of 28/20 °C day/night temperature, 14/10 h light/dark photoperiod, 800 μmol m^−2^ s^−1^ light intensity and 60% humidity. Two-week-old plants were sprayed with 50 μM ACC (or a mock solution without ACC) on the leaf, irrigated with 2 l of hormone solution in a plastic case and then incubated for 5 h before root tissue collection.

For flooding stress treatment, two-day-old seedlings cultivated on quartz sand were completely submerged in 700 ml of water for 5 days at 25 °C with a light/dark cycle (600 μmol m^−2^ s^−1^, 16 h light/8 h dark). The water level was kept at 2 cm above the quartz sand surface, and control seedlings were grown with a water level below the quartz sand surface. Roots and hypocotyls were collected from soybean seedlings. The collected tissues were frozen immediately in liquid nitrogen and stored at −80 °C. All samples were collected in biological triplicate.

For waterlogging treatment, transgenic and control seeds were sown in cones filled with turface and sand in the ratio of 2:1. The cones with plants at the V1 stage were kept inside a tub and flooded above the soil level (>2 cm high) for 14 days. The greenhouse temperatures (24~26 °C) were exactly the same during day and night. The heating system turned on when temperatures were below 23.8 °C. A passive cooling ridge vent opened when temperatures were above 26.6 °C. Active cooling fans turned on when temperatures were above 29.4 °C. The shade was set to stay open all the time, and the HID lights were set to be on at all levels between 5 am and 7 pm.

### 4.4. Promoter Analysis

The DNA sequences 2000 bp upstream of the translation start site (ATG) were extracted from the soybean genome, and the presence and abundance of the known cis-elements were analyzed with the help of the program SOGO (available online: https://sogo.dna.affrc.go.jp/cgi-bin/sogo.cgi?lang=en&pj=640&action=page&page=newplace).

### 4.5. Expression Profiling Using RNA-seq Datasets

The RNA-seq data generated by Libault et al. [32] from nine different soybean tissues (William 82 genotype) including flowers, leaves, nodules, pods, roots, root hairs, seeds, shoot apical meristems, and stems were used to analyze expression patterns of *GmXTH*s members. Chen et al. [34] generated RNA-seq data using soybean (Williams 82 genotype) leaf tissue under flooding stress. Briefly, flooding stress was imposed at the soybean V4 stage (four unfolded trifoliate leaves) by placing the pots into a larger pot filled up to a water level of 4 cm above the soil surface for 7 days.

### 4.6. RNA Extraction for Expression Pattern Analysis

The frozen samples were ground to powder in liquid nitrogen with a mortar and pestle. Approximately 100 mg tissue samples were used for RNA extraction using a RNeasy Plant mini kit (Cat# 74904, Qiagen, Valencia, CA, USA) according to the manufacturer’s protocol. On-Column DNA digestion performed by following the RNase-Free DNase kit (Cat#79254, Qiagen) manufacturer’s protocol. The quality and quantity of RNA were assessed using a Nanodrop® 1000 spectrophotometer (Thermo Scientific, Wilmington, DE, USA).

### 4.7. Quantitative RT-PCR Analysis

A total of 2 µg RNA from each sample was reverse-transcribed to cDNA in 20 µL reaction volume using RNA to cDNA Ecopry™ Premix (Double primed) cDNA Synthesis Kit (Cat# 639549, Clontech, Foster City, CA, USA) according to the manufacturer’s protocol. PCR was performed in a 10 µL reaction volume using the Maxima SYBR Green/ROX qPCR Master Mix (Cat# K0223, Thermo, USA) on the ABI7900HT detection system machine (ABI PRISM^®^ 7900HT, Foster City, CA, USA). The results from three biological replicates and two technical replicates were used for data analysis. The PCR conditions were as follows: 50 °C for 2 min, 95 °C for 10 min, and 40 cycles of 95 °C for 15 s, and 60 °C for 1 min. To normalize the gene expression levels, the actin (Glyma.18G290800) gene was used as an internal control. All novel primers were designed using the Primer3 web interface (available online: http://frodo.wi.mit.edu/primer3/ [63,64]. The primer sequences are listed in Supplementary Table S2.

### 4.8. Construction of the pZY101-AtXTH31 Vector, Agrobacterium-mediated Soybean (Glycine max) Transformation and Progeny Segregation Analysis

The gene-specific primer pair 5′-CATGCCATGGATGGCTTTGTCTCTTATCTTTC-3′ and 5′-CATGCCATGGCTAACATTCTGGTGTTTGGG-3′ was designed to isolate the full-length CDS of *AtXTH31* from *Arabidopsis*. The PCR product (902 bp) was cloned into the pCR4-TOPO vector, and the positive plasmid was fully sequenced with M13 sequencing primers. The *AtXTH31* gene sequence was inserted into the pCNSH.131. AtMyb2p-Gus vector, which contained the Myb2 promoter. Finally, the whole cassette contained a promoter, and the gene sequence was moved into the pZY101-Asc binary vector. An improved *Agrobacterium*-mediated transformation of the soybean cotyledonary node system [65] was performed using the elite genotype “Maverick”. To determine the segregation of gene of interest and selectable marker gene, at least 30 plants from each T0 event were screened using leaf paint (100 mg/L glufosinate, Sigma, St. Louis, MO, USA) analysis carried out for the T0 generation. T2 progeny from the T1 generation was similarly analyzed to identify homozygous T1 lines for subsequent study.

### 4.9. DNA Extraction and Quantification and PCR Confirmation of Transgenes

DNA was extracted from the transgenic plant leaves (mixed leaf tissue) using CTAB methods [66]. DNA concentrations and quality were initially estimated using a Nanodrop spectrometer (Thermo Fisher Scientific, Waltham, MA, USA) and then estimated using a QuantiT dsDNA HS Kit (Invitrogen, Thermo Fisher Scientific, Waltham, MA, USA). The concentrations from the Qubit assays were used to quantify the DNA input in each PCR reaction. Primers were designed to detect the *AtXTH31* gene of interest and the bar gene as the selectable marker gene (Supplementary Table S2). PCR conditions were as follows: 95 °C hot start 30 s, followed by 36 cycles of 95 °C denaturing 10 s, 55 °C annealing 10 s, and 72 °C extension 1 min, followed by 72 °C final extension 10 min. PCR products were analyzed on an agarose gel, and events were considered transgenic if they displayed an approximately 800 bp band for the gene of interest and a 500 bp band for the bar gene. Four positive transgenic events were obtained and used for further phenotype analysis.

### 4.10. TaqMan Assays and QuantStudio 3D Digital PCR Analysis for Soybean AtXTH31 Transgenic Copy Number Variation

The following equipment and chemicals were used from Applied Biosystems (Waltham, MA, USA), Thermo Fisher Scientific, USA: QuantStudio™ 3D Imager (Cat#: PN4489084), QuantStudio™ 3D Loader (Cat#: PN4482592), Dual Flat Block GeneAmpR PCR System 9700 (Cat#: PN4428235), Tilt Base & chip adapters (Cat#: PN4486414 and 4485513), QuantStudio™ 3D Digital PCR 20K Chips (Cat#: PN4485507), and QuantStudio™ 3D Digital PCR Master Mix (Cat#: PN4485718). The probe was designed and synthesized by Life Technology Company. The dPCR reaction volume was 20 µL and contained 10 µL 2× TaqMan 3D mix, 1 µL 20× FAM labeled primers and probe, 1 µL 20× VIC labeled primers and probe, 1 µL DNA samples (40 ng/ µL), and 7 µL nuclease-free water. In total, 14.5 µL each reaction product was loaded on the chips. Data analysis was conducted using QuantStudio™ 3D Analysis Suite™ Cloud Software as described previously [55]. The designed probes could only amplify transgene *AtXTH31* to ensure that no soybean homologous genes were detected. The ratio of the copy number of *AtXTH31* with lectin in the same soybean material was calculated as follows: (copies/µL of the *AtXTH31* transgene)/(copies/µL of the lectin gene Glyma.02G009600) in the same PCR reaction product. Soybean transgenic plants contained a single insert copy when the ratio value was equal to 0.5 and two insert copies when the ratio value was equal to 1. The ratio value was less than 0.5 when chimeric transgenic plants were found. The primers and probe sequences are shown in Supplementary Table S2.

## 5. Conclusions

In conclusion, our results showed that the soybean genome contains 61 *XTH* genes, the largest family of XTH proteins characterized in any organism to date. The results of phylogenetic analysis and chromosome location/structure provide an overview of the soybean *GmXTH* gene family. The results of the segmental and tandem duplication during expansion of the *GmXTH* gene family provide a genome-wide evolutionary overview. The results of conserved amino acid motif analysis and expression pattern analysis further provide insight into their putative function. Additionally, functional analysis of *AtXTH31* in a heterologous system suggests that the higher germination rate and longer roots/hypocotyls induced by the increased XTH activity may be responsible for the flooding tolerance of transgenic plants. Further comprehensive experiments may be required to elucidate the cellular locations and functions to understand the biological role of XTHs in soybean.

## Supplementary Materials

Supplementary materials can be found at http://www.mdpi.com/1422-0067/19/9/2705/s1. Supplementary Table S1: Information related to the 61 genes homologous to XTH genes in the soybean genome; Supplementary Table S2: Oligonucleotide primer sequences used in this work; Supplementary Table S3: Estimate of the dates of the segmental duplication events of the soybean XTH gene pairs; Supplementary Figure S1: Exon-intron structure of soybean *XTHs*; Supplementary Table S4: Search for cis-elements in the GmXTH gene promoters.
