# Supplementary material for: Characterization of the XTH Gene Family: New Insight to the Roles in Soybean Flooding Tolerance
Source: Int J Mol Sci. 2018 Sep 11;19(9):2705. doi: 10.3390/ijms19092705 (PMC6164600; doi:10.3390/ijms19092705)
Supplement: Supplementary file 1 [file ijms-19-02705-s001.zip › ijms-327328 supplementary/Supplementary Figure 1¿CGene structure.pdf]

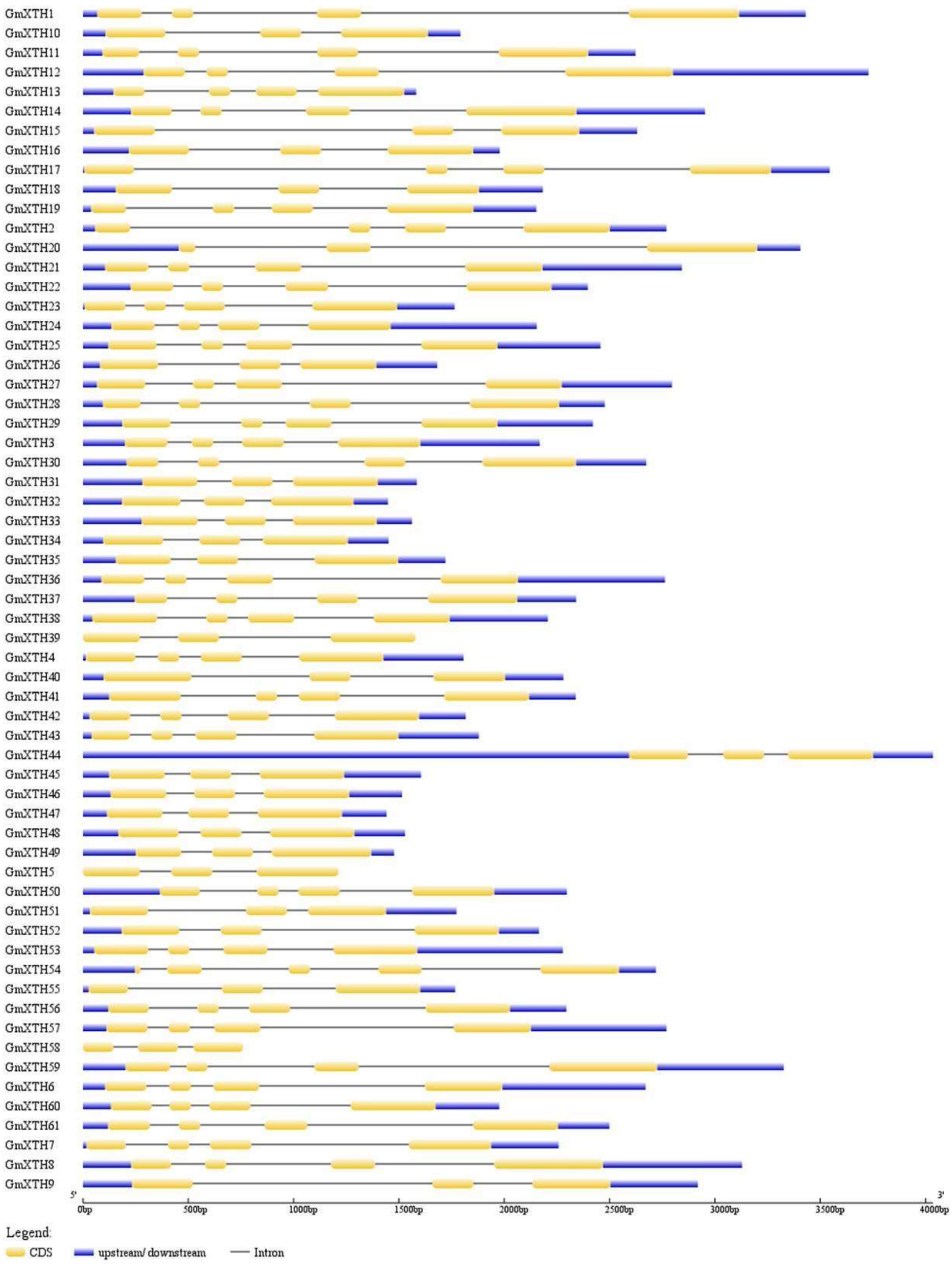

Supplementary Figure 1. Exon-intron structure of soybean XTHs. The lengths of the exons and introns of each XTH gene are displayed proportionally. Exons and introns are indicated by yellow rectangles and thin lines, respectively. The untranslated regions (UTRs) are indicated by blue rectangles.
